# The Sleep Puzzle: Linking Glymphatic Function to Cognitive Decline

**DOI:** 10.1111/ene.70451

**Published:** 2025-11-26

**Authors:** Matti Einari Ahlström, Aleksi Johannes Sihvonen

**Affiliations:** ^1^ Department of Neurology Helsinki University Hospital and University of Helsinki Helsinki Finland

**Keywords:** cerebral small vessel disease, cognitive impairment, glymphatic system, neuroradiology, sleep

It has been almost 200 years since the first medical description of cerebral small vessel disease (cSVD). Beginning in the 1830s, several French physicians, most notably Maxime Durand‐Fardel and Pierre Marie, reported distinct clinicopathological observations of patients with cognitive decline, gait disturbances and stroke episodes. Marie, appearing almost prescient, associated his patients' clinical symptoms with what would later become the radiological hallmarks of cSVD—lacunar infarcts, enlarged perivascular spaces, white matter tissue abnormalities and atrophy, as well as intracerebral haemorrhaging [1].

Despite this rich history, cSVD remains a global health concern and a major cause of cognitive impairment, with recent ESO guidelines highlighting the scarcity and generally low‐quality evidence to support existing therapeutic interventions [2]. Additionally, the pathophysiology of cSVD has remained elusive, with earlier models of chronic hypertensive hypoperfusion‐induced ischemia appearing too rigid to account for the apparent heterogeneity among cSVD patients. Neurovascular unit dysfunction, blood–brain barrier leakage, inflammation and oligodendrocyte dysfunction have all been implicated in the cellular‐level pathogenesis of cSVD [1].

The discovery of the glymphatic system in 2012 introduced a striking new viewpoint to the debate on brain health. A fluid and waste clearance system in the brain that had previously been completely hidden from pathologists, radiologists and clinicians alike? The notion seemed as preposterous as it was exciting. The glymphatic system has been proposed to permeate the brain parenchyma utilising perivascular spaces and has been speculated to be defective in a variety of neurological disorders [3].

As the glymphatic pathways appear to be most active during deep sleep, there has been renewed interest in assessing sleep quality not only as an indicator of subjective well‐being, but as a predictor of later neurodegeneration [4]. This seems especially pertinent with cSVD, as the major anatomical features involved in the glymphatic system are also areas of impairment in cSVD. It has indeed recently been proposed that glymphatic dysfunction and sleep disturbances can be contributing factors in the pathology of cSVD [3].

While most of our data on the glymphatic system comes from rodent models, waste removal certainly seems like an obvious and important element of maintaining health through senescence in the mammalian brain. Could a lack of deep, cleansing sleep be the missing link to explain why some patients with similar risk factors develop neurodegenerative disorders like Alzheimer's disease, while others don't? It has already been demonstrated that a single night of sleep deprivation impedes molecular clearance in the human brain, with the contrast marker used interpreted by the authors as a surrogate for amyloid‐beta and tau [5]. The truth, most probably, is not as simple as that.

While the animal model of the glymphatic system has been well documented, studies in humans have been limited due to the lack of non‐invasive imaging techniques [3] (after all, not every research subject can be asked to undergo repeated intrathecal injections). Recently, diffusion tensor image analysis along the perivascular space (DTI‐ALPS) has been introduced as a method to assess the movement of water molecules in the perivascular spaces [6]. While not a perfect imaging technique to describe the dynamic (and markedly circadian) glymphatic system, DTI‐ALPS can offer us a snapshot of perivascular fluid flow in the human brain.

In this month's issue of the European Journal of Neurology, Zhou et al. [7] report a study linking glymphatic dysfunction, as assessed with DTI‐ALPS, with poor subjective sleep quality and cognitive impairment. Interestingly, high‐risk cSVD subjects with poor sleep quality were more at risk of cognitive impairment, and over a 7‐year follow‐up period, baseline DTI‐ALPS and cSVD burden were significantly correlated with longitudinal cognitive decline, as indexed by decreases in Mini‐Mental State Examination (MMSE) scores. This seems to play into the idea that poor sleep quality can be a major risk factor—or perhaps even a contributing cause—of cSVD progressing towards a more symptomatic phase.

However, the discussion on glymphatic dysfunction is not without controversy. Some have even questioned the existence of the glymphatic system in the human brain, at least subcortically [8]. DTI‐ALPS specifically has come under criticism for possibly overemphasising subcortical involvement in perivascular cerebrospinal fluid flow and introducing bias towards positive findings in a variety of disconnected neuropathologies [8]. Additionally, as discussed above, DTI‐ALPS offers only a static glimpse into a very dynamic system.

Nevertheless, the possible shortcomings of the DTI‐ALPS method have been previously addressed by fine‐tuning the imaging technique with a modified scale [9]. This modified scale has also been used in the article under discussion, where the veracity of the DTI‐ALPS findings was reproduced using the modified DTI‐ALPS index (mDTI‐ALPS), with the results holding firm (the authors have kindly supplied this information in Appendix S1); [7].

Given that we have not found a suitable imaging technique for the glymphatic system, even after over a decade since its discovery, wouldn't it be prudent to simply wait for imaging technology to catch up? Perhaps. However, the basic tenet of the scientific method is to pursue new information where that is reproducibly available. DTI‐ALPS has shown itself to be reproducible [9], and at the moment seems to be an important, non‐invasive tool we can use to describe a very complex system that we are just beginning to understand. Additionally, it is worth noting that DTI‐ALPS is only one tool at our disposal, with others being developed in unison [10].

We are seeing pieces of a puzzle everywhere, with some denoting a physiological phenomenon of our brains, hitherto hidden in the deep, glial‐lymphatic contours of our parenchyma. Other puzzle pieces are describing the centuries‐old pathological conditions of SVD, Alzheimer's disease, Parkinson's disease and a myriad of other ailments. Perhaps, after a time, we can step back and look at the puzzle we are constructing. It very well may be, that all this time radiologists, physiologists, anatomists and clinicians have been working on the same puzzle. Perhaps our corner pieces will be matching, and the whole puzzle will describe a unified view of sleep‐related neurodegenerative pathology. After all, we are such stuff as dreams are made on, and our little life is rounded with a sleep [11].

## Author Contributions

**Matti Einari Ahlström:** conceptualization, writing – original draft, writing – review and editing. **Aleksi Johannes Sihvonen:** writing – review and editing.

## Conflicts of Interest

The authors declare no conflicts of interest.

## Supporting information


**Appendix S1:** ene70451‐sup‐0001‐AppendixS1.pdf.

## Data Availability

The authors have nothing to report.
