# Supplementary material for: The Sleep Puzzle: Linking Glymphatic Function to Cognitive Decline
Source: Eur J Neurol. 2025 Nov 26;32(12):e70451. doi: 10.1111/ene.70451 (PMC12658283; doi:10.1111/ene.70451)
Supplement: Supplementary file 1 — Appendix S1: ene70451‐sup‐0001‐AppendixS1.pdf. [file ENE-32-e70451-s001.pdf]

Wiley **#2929422**

Ticket Details

Status	Priority	Source	Type
Closed	Low	Email	
**Group**	**Agent**	**Journal**	**Type (Wiley Peer Review)**
Production Editors (SPS)	Virgil Rozita	ENE	
**Level 2**	**Level 3**	**Pending Status**	**Type**
**Reason**	**SAP Journal Acronym**	**SAP PO**	**Material**
**GPOM Type**	**Licensing Type**	**Non-Wals OA Request**	**Estimating/AA Type**
**Mail With**	**Supplement**	**Version(s)**	**TL**
			Appavoo, Raymond
**PM**	**SPM**	**GPOM Type (Notification)**	**GPOM Type (Incident)**
Peer Mohamed, Kuthsiyya	Lau, Michelle		
**Team**	**Material (2)**	**PBO submission**	**Delivery date**

by **Matti. ahlstrom** on **Mon, 24 Nov at 3:20 PM** via **Email**

VS: Communication related to article: ENE_70451

Greetings,

The reference for Zhou reads as follows: "Zhou R, Zhong W, Xia Y, Ying Y, Wang Y, Yang L, Xu J, Cao J, Liang Z, Wang X, Dong Q, Ding D, Cheng X. The Relationship Between Cerebral Small Vessel Disease, Sleep Quality, and Cognitive Impairment Among Community-Dwelling Older Adults: Exploring the Role of Glymphatic Function. Eur J Neurol. 2025 Oct;32(10):e70384"

As I understand it, both of the queries can refer to this reference.

Regards,
Matti

Lähettäjä: ENE@wiley.com <ENE@wiley.com>
Lähetetty: maanantai 24. marraskuuta 2025 11.12
Vastaanottaja: Ahlström Matti <Matti.Ahlstrom@hus.fi>

Kopio: bpcorrections@straive.com

Aihe: Communication related to article: ENE_70451

Et saa usein sähköpostia osoitteesta ene@wiley.com. Lue, miksi tämä on tärkeää

Dear author,

Thank you for supplying the corrections for the article. Kindly provide your response to the attched query.

Regards,

Virgil

The contents of this email and any attachments are confidential and intended only for the person or entity to whom it is addressed. If you are not the intended recipient, any use, review, distribution, reproduction or any action taken in reliance upon this message is strictly prohibited. If you received this message in error, please immediately notify the sender and permanently delete all copies of the email and any attachments.

Comments

by **Virgil Rozita** on **Mon, 24 Nov at 5:52 PM** as **Outbound email**

Dear author,

Thank you for your email, I will proceed further with inserting the reference.

Best regards,

Virgil Rozita
She/Her
Production Editor
WILEY
